# On the distribution of cycles and paths in multichromosomal breakpoint graphs and the expected value of rearrangement distance

**DOI:** 10.1186/1471-2105-16-S19-S1

**Published:** 2015-12-16

**Authors:** Pedro Feijão, Fábio Viduani Martinez, Annelyse Thévenin

**Affiliations:** 1 Faculty of Technology, Bielefeld University, Universitätsstraße 25, 33615 Bielefeld, Germany; 2 Institute for Bioinformatics, Center for Biotechnology (CeBiTec), Bielefeld University, Bielefeld, Germany; 3 Faculdade de Computação, Universidade Federal de Mato Grosso do Sul, 79070-900 Campo Grande, Brazil

**Keywords:** Hultman number, multichromosomal genomes, genome rearrangement, breakpoint graph, double cut and join

## Abstract

Finding the smallest sequence of operations to transform one genome into another is an important problem in comparative genomics. The breakpoint graph is a discrete structure that has proven to be effective in solving distance problems, and the number of cycles in a cycle decomposition of this graph is one of the remarkable parameters to help in the solution of related problems. For a fixed *k*, the number of linear unichromosomal genomes (signed or unsigned) with *n *elements such that the induced breakpoint graphs have *k *disjoint cycles, known as the *Hultman number*, has been already determined. In this work we extend these results to multichromosomal genomes, providing formulas to compute the number of multichromosal genomes having a fixed number of cycles and/or paths. We obtain an explicit formula for circular multichromosomal genomes and recurrences for general multichromosomal genomes, and discuss how these series can be used to calculate the distribution and expected value of the rearrangement distance between random genomes.

## Background

In molecular biology and genetics, comparative genomics is a discipline interested in the comparison of genomic attributes of different organisms. These attributes may encompass DNA sequences, gene content, gene order, regulatory sequences, and other structural features. Several measures have been proposed to compute the (dis)similarity between genomes. The field called *genome rearrangements *is concerned with measures of dissimilarity involving large-scale mutations, such as reversals and transpositions, where a fundamental problem is to determine the smallest sequence of such rearrangement operations that transforms one given genome into another. This minimum number of operations is called the *rearrangement distance *between the two given genomes. These and other aspects of genome rearrangements are discussed in detail by Fertin *et al*. [1].

A remarkable characteristic of methods to compute distances is the systematic use of a graph, first introduced by Bafna and Pevzner [2], known as the *breakpoint graph*. It has proven, by its decomposition into disjoint cycles, a useful tool to efficiently compute rearrangement distances such as transposition or reversal, directly related to the number of cycles in this decomposition [1].

Since cycle decomposition of breakpoint graphs plays a central role in computing distances, it is useful to investigate the distribution of such cycles. Particularly, the distribution of genomes with a number of cycles *c *allows us to evaluate the probability to have a scenario of a distance *d *depending of *c*. Doignon and Labarre [3] enumerated the unsigned permutations of a given size such that the corresponding graph has a given number of cycles, and called it the *Hultman number*. Subsequently, Grusea and Labarre [4] extended this result for *signed *permutations, where the signs model gene orientation.

In this work we extend previous results providing formulas to compute the number of multichromosomal genomes with a given number of cycles and/or paths. We obtain an explicit formula for circular genomes and recurrences for more general cases.

Our paper is organized as follows. In the Preliminaries section we give some definitions and notations. The results for circular and general multichromosomal genomes are presented in the next section, called The Multichromosomal Hultman Number. The following section presents some discussion about the distribution of the rearrangement distance, derived from the multichromosomal Hultman numbers, and the Conclusion section presents final remarks and perspectives.

## Preliminaries

We represent multichromosomal genomes using a similar notation as in [5]. A *gene *is a fragment of DNA on one of the two DNA strands in a chromosome, showing its orientation. A gene is represented by an integer and its orientation by a sign. The orientation of a gene *g *allows us to distinguish its two *extremities*, the *tail *(*g^t^*) and the *head *(*g^h^*). A *chromosome *is represented by a sequence of genes, flanked in the extremities by *telomeres *(∘) if the chromosome is linear; otherwise, it is circular. *Genomes *are represented as sets of chromosomes. An *adjacency *in a genome is either a pair of consecutive gene extremities in a chromosome, or a gene extremity adjacent to a telomere (a *telomeric adjacency *). For instance, *A *= {(∘ 1 2 3 4 ∘)} is a genome with one linear chromosome and four genes, and has the adjacencies ∘1*^t^*, 1*^h^*2*^t^*, 2*^h^*3*^t^*, 3*^h^*4*^t ^*and 4*^h^*∘, where the first and the last are telomeric adjacencies.

There is a one-to-one correspondence between genomes and *matchings *in the set of extremities. Adjacencies correspond to two matched (saturated) vertices, and telomeric adjacencies correspond to unmatched (unsaturated) vertices. Therefore, a perfect matching (i.e., matching which saturates all vertices of the graph) corresponds to a genome with only circular chromosomes. The matching corresponding to a genome *A *is denoted by *M_A_*. Because of this one-to-one relationship, in this text we use the terms *genome *and *matching *interchangeably.

**Figure 1:**
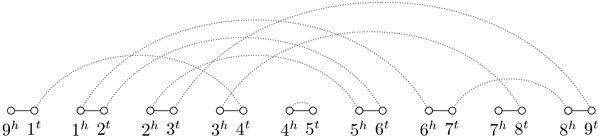
**Multichromosomal breakpoint graph**. Input genomes are *A *= {(1 2 3 4 5 6 7 8 9)} (black edges) and *B *= {(6 -1 4 5 -2), (∘ -9 3 8 7 ∘)} (dotted edges).

Given two genomes *A *and *B *with the same set of genes, the *multichromosomal breakpoint graph *of *A *and *B*, denoted by *BG*(*A, B*), is built by joining the matchings *M_A _*and *M_B _*in the same set of vertices, using different colors for the edges of each matching. Figure 1 shows an example of a multichromosomal breakpoint graph for genomes *A *= {(1 2 3 4 5 6 7 8 9)} and *B *= {(6 -1 4 5 -2), (∘ -9 3 8 7)}. From this point on we will use the term *breakpoint graph *to refer to the multichromosomal breakpoint graph. Since all its vertices have degree 0, 1 or 2, the breakpoint graph is uniquely decomposed in cycles and paths. For instance, the breakpoint graph in Figure 1 is decomposed in two cycles and one path.

## The multichromosomal Hultman number

In this section, we extend the results of [3,4] for multichromosomal genomes. There are two new aspects that must be considered. First, since the breakpoint graph can be decomposed in cycles and paths, we may have to count not only cycles, but also paths. The other question is about the *identity genome*. In the unichromosomal case, the identity genome is easily defined. In the multichromosomal case, it is not obvious which given genome is the identity. When working on multichromosomal circular genomes, the identity is defined as in the unichromosomal case. In the general case, working on genomes with linear and circular chromosomes, we analyze two types of identities for genomes: one with only one set of circular chromosomes and another with a set of circular chromosomes and a set of linear chromosomes.

In the next sections, we propose extensions of the Hultman number for multichromosomal genomes, first considering only circular genomes, and then extending the results to general genomes, with linear and circular chromosomes. The same strategy is used in all cases: first, start with a matching representing the identity, and then superimpose all other possible matchings, while counting recursively cycles and paths. To do that, we need to consider all possible operations to build such matchings. In Figure 4, all such operations are shown.

### Multichromosomal circular genomes

A *circular genome *is a genome where all chromosomes are circular. Since there are no telomeric adjacencies, the matching *M_A _*of a circular genome *A *is a perfect matching on the extremities of *A*. Moreover, the breakpoint graph of two circular genomes is decomposed in disjoint alternating cycles, since each vertex has degree two.

We want to compute the number of circular genomes with *n *genes that have *c *disjoint alternating cycles over a given identity genome *I*, that we call the *multichromosomal circular Hultman number*, denoted by *H_C _*(*n, c*). In this case, since the matching of any circular genome is a perfect matching, we claim that *H_C _*(*n, c*) is the same, independently of the genome *I *chosen as an identity, and simply define *I*_∘ _= {(1, 2,..., *n*)}. Hence, we define

(1) $$H_{C}\left(n,\;c\right)\equiv |\left\{A\in C_{n}:cyc\left(BG\left(A,\;I_{\circ }\right)\right)=c\right\}|,$$

where $C_{n}$ is the set of all circular multichromosomal genomes with *n *genes and *cyc*(*G*) denotes the number of cycles in a graph *G*.

Starting with a perfect matching $M_{I_{\circ }}$ of the 2*n *vertices, we build all breakpoint graphs *BG*(*A*, *I_∘_*), for circular genomes *A*, which correspond to perfect matchings, adding one edge at a time, while counting the number of cycles, recursively.

**Figure 2:**
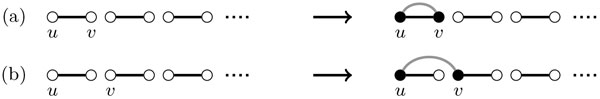
**Construction of the breakpoint graph for a circular identity genome *I *and a circular genome *A***. The adjacencies of *I *are represented by black edges and those of *A *by grey edges. Unvisited nodes are white, visited ones are black. To build a perfect match (circular genome *A*) only two operations are possible: (a) Create a cycle; (b) Merge two paths.

The matching $M_{I_{\circ }}$ is composed by *n *connected components, and all are paths. Considering an arbitrary vertex *u *in the matching $M_{I_{\circ }}$, there are 2*n *- 1 possible edges *uv *that can be created. Figure 2 shows how these different edges can be chosen. There are two possible cases:

**(a) Create Cycle**: If *u *and *v *belong to the same component, the edge *e *= (*u, v*) will *create a cycle*. There is only one possibility for this type of edge.

**(b) Merge Paths**: If *u *and *v *belong to different components, *uv *will *merge both paths*. There are 2*n *- 2 possibilities of adding such an edge.

Applying any of the two operations results in a graph with *n *- 1 paths, a subcase of the original graph with *n *paths, with operation (a) also creating a cycle. This allows us to establish a recurrence for *H_C _*(*n, c*). For the base cases, when *n *= 0 we only have the empty genome, with 0 cycles in the breakpoint graph. Therefore, *H_C _*(0, *c*) = 1 if and only if *c *= 0, with *H_C _*(0, *c*) = 0 for *c *> 0. Also, if either *n *or *c *is less than zero, we have that *H_C _*(*n, c*) = 0.

$$H_{C}\left(n,\;c\right)=\left\{\begin{array}{cc} 0, & \text{if}\;n=0\;\text{and}\;c>0,\\ 0, & \text{if}\;n<0\;\text{or}\;c<0,\\ 1, & \text{if}\;n=c=0,\\ H_{C}\left(n-1,\;c-1\right)+\left(2n-2\right)\cdot H_{C}\left(n-1,\;c\right), & \text{if}\;n>c. \end{array}\right)$$

The following result states an explicit formula to *H_C _*(*n, c*).

Theorem 1

$$H_{C}\left(n,c\right)=\frac{2^{n-c}}{\left(c-1\right)!}\underset{\begin{array}{c} 0\leq q_{1},\ldots ,q_{n-c}:\\ \sum_{2}^{n-c}mq_{m}=n-c \end{array}}{\sum }\frac{\left(n+Q-1\right)!}{q_{2}!\cdots q_{n-c}!1!q_{1}2!q_{2}\ldots k!q_{n-c}},$$

*where Q *= *q*_2 _+ ... + *q_k _and *$\sum_{2}^{n-c}mq_{m}=n-c$*is a sum over all partitions of n - c*.

*Proof *We know from [6] that unsigned Stirling numbers of first kind satisfy the following recurrence equation: $\left[\begin{array}{c} n\\ c \end{array}\right]=\left[\begin{array}{c} n-1\\ c-1 \end{array}\right]+\left(n-1\right)\left[\begin{array}{c} n-1\\ c \end{array}\right]$. Multiplying both sides by 2*^n-c ^*and using *H_C _*(*n, c*) recurrence equation we arrive at $H_{C}\left(n,\;c\right)=2^{n-c}\left[\begin{array}{c} n\\ c \end{array}\right]$. Then, using the explicit formula for $\left[\begin{array}{c} n\\ c \end{array}\right]$ given in [7], we arrive at our result.   □

Furthermore, the sequence of integers generated by *H_C _*(*n, c*) is the unsigned entry A039683 in the OEIS (On-Line Encyclopedia of Integer Sequences) [8].

### General multichromosomal genomes

We will generalize our previous formula for general multichromosomal genomes, with both linear and circular genomes. As already mentioned, two difficulties arise. Now, we have not only cycles but also paths in the breakpoint graph. Thus, it is not clear which genome should be considered the identity genome. As a starting point, let us consider again the identity as *I*_∘ _= {(1, 2,..., *n*)}, and find the *general Hultman number H_G_*(*n, c, p*), defined as

(2) $$H_{G}\left(n,c,p\right)\equiv |\left\{A\in G_{n}:cyc\left(BG\left(A,\;I_{\circ }\right)\right)=c\;\text{and}\;pt\left(BG\left(A,\;I_{\circ }\right)\right)=p\right\}|,$$

where $G_{n}$ is the set of all multichromosomal genomes with *n *genes, and *pt*(·) denotes the number of paths in a graph. In this set, each genome corresponds to a matching, not necessarily perfect, since only circular genomes correspond to perfect matchings. Similarly as the previous case, we start with the matching $M_{I_{\circ }}$ on 2*n *vertices, and recursively build all possible matchings, while counting cycles and paths. Since a matching induced by an arbitrary genome *A *in $G_{n}$ is not necessarily perfect, together with the *create cycle *and *merge paths *operations on a vertex *u*, we can also choose to not saturate a vertex *u *in the matching being built, thus creating a telomere, which we call a *skip vertex *operation.

Moreover, since we now have an operation that is applied on just one vertex, and not two at a time such as the operations presented in Section, we need to define a different recurrence, where *n *correspond to vertices in the breakpoint graph, and not to genes in the genomes. In a genome *I*_∘ _with *n *genes, there are 2*n *vertices (extremities) in $M_{I_{\circ }}$ and consequently in *BG*(*A, I*_∘_). So, we need an auxiliary number $H_{G}^{\prime }\left(e,\;c,\;p\right)$, such that $H_{G}\left(n,\;c,\;p\right)=H_{G}^{\prime }\left(e,\;c,\;p\right)$, with *e *= 2*n*, and $H_{G}^{\prime }\left(e,\;c,\;p\right)\equiv |\left\{M\in M_{e}:cyc\left(BG\left(M,\;M_{I_{\circ }}\right)\right)=c\;\text{and}\;pt\left(BG\left(M,\;M_{I_{\circ }}\right)\right)=p\right\}|$, where $M_{e}$ is the set of all possible matchings on *e *vertices, and $M_{I_{\circ }}$ is a perfect matching with *e*/2 edges induced by *I*_∘_.

**Figure 3:**
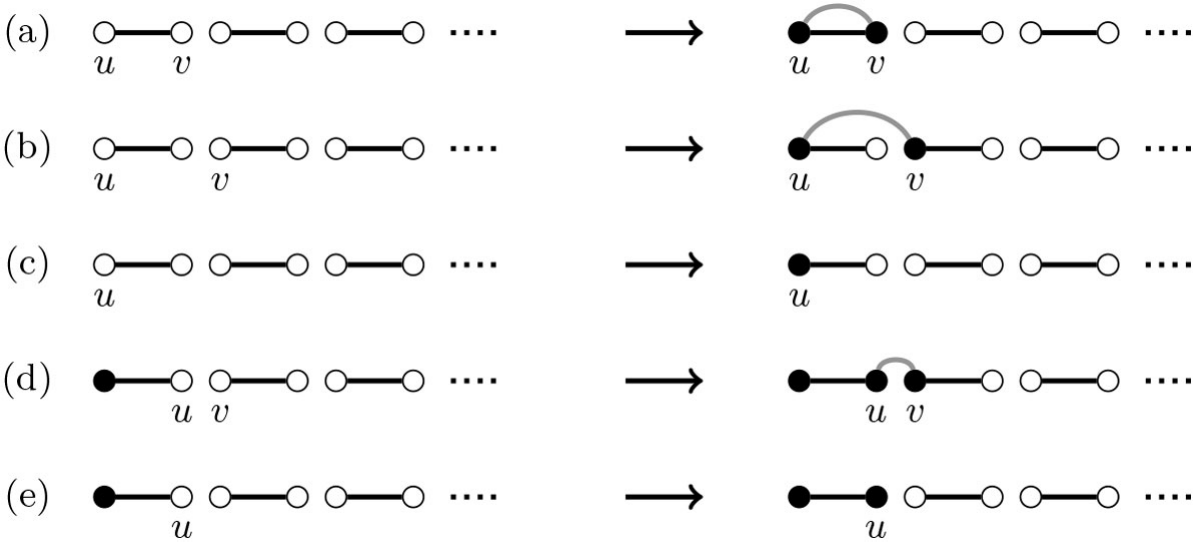
**Construction of the breakpoint graph for a circular genome *I *and a general genome *A***. The adjacencies of *I *are represented by black edges and those of *A *by grey edges. Unvisited nodes are white, visited ones are black. We can create a cycle only when *e *(the number of unvisited nodes) is even (a). We can merge two paths when *e *is even (b) or odd (d). We can skip a vertex when *e *is even (c) or odd (e). In (c) and (d), the parity of the number of unvisited vertices is changed.

Starting with the matching $M_{I_{\circ }}$, another matching is built recursively by adding edges or skipping vertices until all vertices have been *visited*. Visited vertices are shown in figures as black vertices, and *unvisited *as white. If *e *is even, we pick any unvisited vertex *u *and we have tree possibilities (Figure 3a,b,c):

**(a) Create Cycle**: There is one edge *uv *such that *v*(≠ *u*) is the unvisited vertex in the same component as *u*, and this edge (shown as a grey edge *uv*) will create a cycle. Vertices *u *and *v *are marked as visited (Figure 3(a)).

**(b) Merge Paths**: There are *e - *2 edges *uv *such that *v *is an unvisited vertex in a different component as *u*, and this edge will merge these components, that are paths. Vertices *u *and *v *are marked as visited. (Figure 3(b)).

**(c) Skip Vertex**: Vertex *u *is not saturated; no edge is created and only *u *is marked as visited (Figure 3(c)).

If *e *is odd, it means that there is a vertex *u *that is connected to a visited vertex. For this vertex, there is no way to close a cycle, but the other two operations are possible:

**(d) Merge Paths**: There are *e - *1 edges *uv *such that *v *is in a different component as *u*, merging these components. Vertices *u *and *v *are marked as visited (Figure 3(d)).

**(e) Skip Vertex**: Vertex *u *is not saturated; no edge is created, only *u *is marked as visited. A path where all vertices are visited is created (Figure 3(e)).

For the base cases, again we know that when *e *= 0, we have only the empty genome, and this means that $H_{G}^{\prime }\left(0,\;c,\;p\right)=1$ if an only if *c *= *p *= 0, and $H_{G}^{\prime }\left(0,\;c,\;p\right)=0$ if *c *> 0 or *p *> 0. Also, if any of *e*, *c*, or *p *is negative, $H_{G}^{\prime }\left(e,\;c,\;p\right)=0$. With that, we arrive at the following recurrence:

$$H_{G}^{\prime }\left(e,\;c,\;p\right)=\left\{\begin{array}{cc} 0, & \left(1\right)\\ 1, & \left(2\right)\\ {H^{\prime }}_{G}\left(e-2,\;c-1,\;p\right)+\left(n-2\right)\cdot {H^{\prime }}_{G}\left(e-2,\;c,\;p\right)+{H^{\prime }}_{G}\left(e-1,\;c,\;p\right), & \left(3\right)\\ \left(n-1\right)\cdot {H^{\prime }}_{G}\left(e-2,\;c,\;p\right)+{H^{\prime }}_{G}\left(e-1,\;c,\;p-1\right), & \left(4\right) \end{array}\right)$$

with (1) if any of *e, c, p *is negative, or *e *= 0 and any of *c, p *is positive; (2) if *e *= *c *= *p *= 0; (3) if *e *is even; and (4) if *e *is odd.

### Multichromosomal genomes with a fixed number of linear chromosomes

In this section we generalize the previous approach for different identity genomes. Instead of fixing the identity as a circular genome, the identity *I_ℓ _*is a genome with a fixed number of *ℓ *linear chromosomes. As for the input genomes, first we consider all possible genomes, and in a second approach also fix the number of linear chromosomes.

#### Identity genome I_ℓ _with ℓ linear chromosomes

**Figure 4:**
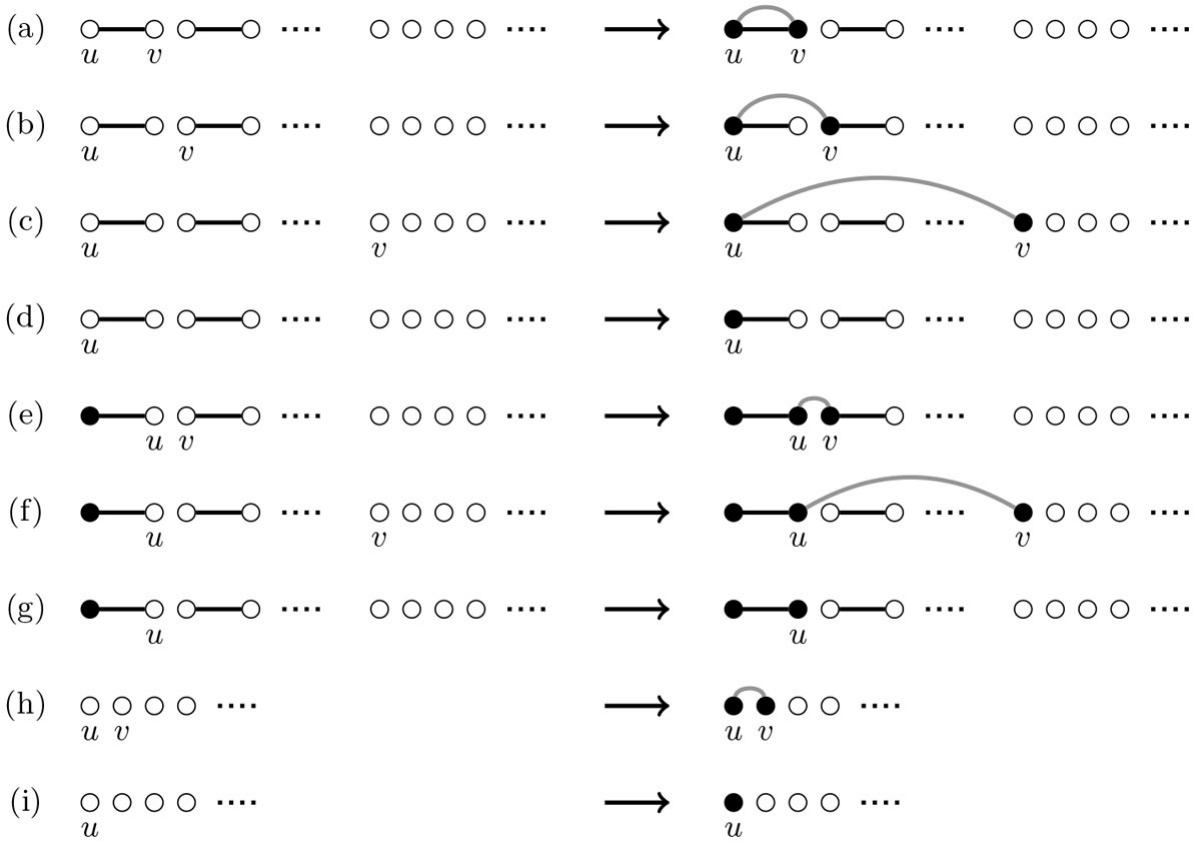
**Construction of matching for genome *I_ℓ _*with *ℓ *linear chromosomes (*i *unsaturated vertices) and a general genome *A***. Adjacencies of *I *are represented by black edges and those of *A *by grey edges. Visited (unvisited) vertices are black (white). We can create a cycle only when *e *+ *i *is even (a). We can merge two paths when *e *+ *i *is even (b) or odd (e). We can connect an unsaturated vertex when *e *= *i *(h), when *e *+ *i *is even (c) or odd (f). We can skip a vertex when *e *= *i *(i), when *e *+ *i *is even (d) or when *e *+ *i *odd (g). In (c) and (d), the parity of *e *+ *i *is changed.

In this case, we can define the Hultman number

(3) $$H_{L}\left(n,\;c,\;p,\;\ell \right)\equiv |\left\{A\in G_{n}:cyc\left(BG\left(A,\;I_{\ell }\right)\right)=c\;\text{and}\;pt\left(BG\left(A,\;I_{\ell }\right)\right)=p\right\},$$

where $G_{n}$ is the set of all multichromosomal genomes with *n *genes, and *I_ℓ _*is a genome with exactly *ℓ *linear chromosomes. This is a generalization of the previous case, since *H_G_*(*n, c, p*) = *H_L_*(*n, c, p*, 0). We propose again an auxiliary series, defined as $H_{L}^{\prime }\left(e,\;c,\;p,\;i\right)\equiv |\left\{M\in M_{n}:cyc\left(BG\left(M,\;M_{I_{i}}\right)\right)=c\;\text{and}\;pt\left(BG\left(M,\;M_{I_{i}}\right)\right)=p\right\}|$, where $M_{n}$ is the set of all possible matchings on *e *vertices, and $M_{I_{i}}$ is a matching on these vertices such that exactly *i *vertices are unsaturated (isolated), with *e *= 2*n *and *i *= 2ℓ. Then, given a matching $M_{I_{i}}$ with *i *unsaturated vertices, we will build a matching recursively adding edges or skipping vertices until all vertices have been visited. In this case, the parity of *e *+ *i *determines which possibilities we have (Figure 4). When *e *+ *i *is even, we will call the current state *balanced*, otherwise it is *unbalanced*. In the balanced case, focusing on an unvisited vertex *u *that is saturated by $M_{I_{i}}$ there are four possible cases (Figure 4a-d):

**(a) Create Cycle**: There is one edge *uv *such that *v*(≠*u*) is an unvisited vertex in the same component as *u*, and this edge will create a cycle. Vertices *u *and *v *are marked as visited.

**(b) Merge Paths**: There are *e - *2 *- i *edges *uv *such that *v *is saturated in *I_i _*and is in a different component as *u*, and *uv *will merge these components, that are paths. Vertices *u *and *v *are marked as visited.

**(c) Skip Vertex**: No edge is created and *u *is marked as visited.

**(d) Connect with unsaturated**: There are *i *possible edges from *u *to an unsaturated vertex *v *in *I_i_*. Vertices *u *and *v *are marked as visited.

Cases (a) and (b) visit two vertices that are saturated in *I_i_*, which means that the state remains balanced. Case (c) changes the state to unbalanced, since only one vertex is visited. Case (d) visits two vertices, but one is a unsaturated vertex in *I_i_*, which means that the parity of *e *+ *i *changes and the state becomes unbalanced.

In the unbalanced state, focusing on a vertex *u *belonging to a component with all other vertices visited, there are three possibilities (Figure 4e-g):

**(e) Merge Paths**: There are *e - *1 *- i *edges *uv *such that *v *is saturated in *I_i _*and is in a different component as *u*, and this edge will merge these components, that are paths. Vertices *u *and *v *are marked as visited.

**(f) Skip Vertex**: Vertex *u *is not saturated in *M *; no edge is created and only *u *is marked as visited, and a path with all vertices visited is created.

**(g) Connect with unsaturated**: There are *i *possible edges from *u *to an unsaturated vertex *v *in *I_i_*. Vertices *u *and *v *are marked as visited, and a path with all vertices visited is created.

Cases (e), (f) and (g) are similar to cases (b), (c) and (d), respectively, which means that (e) keeps the state unbalanced, but (f) and (g) change it to balanced again. There are still two cases to consider, when *e *= *i *(Figure 4h, i).

**(h) Connect two unsaturated**: There are *i - *1 possible edges from an unsaturated vertex *u *to an unsaturated vertex *v *in *I_i_*. Vertices *u *and *v *are marked as visited, and a path with all vertices visited is created.

**(i) Skip Vertex**: No edge is created and *u *is marked as visited. A path with all vertices visited is created.

For the base cases, as before when *e *= 0 we have $H_{L}^{\prime }\left(0,\;c,\;p,\;i\right)=1$ if and only if *c *= *p *= *i *= 0, and $H_{L}^{\prime }\left(0,\;c,\;p,\;i\right)=0$ if any of *c, p, i *is positive. Also, if any of *e, c, p, i *is negative, $H_{L}^{\prime }\left(e,\;c,\;p,\;i\right)=0$.

With all these cases described, we arrive at the recurrence, from what we can deduce *H_L_*(*n, c, p, ℓ*):

$$H_{L}^{\prime }\left(e,\;c,\;p,\;i\right)=\left\{\begin{array}{cc} 0, & \left(1\right)\\ 1, & \left(2\right)\\ \begin{array}{c} \left(i-1\right)\cdot {H^{\prime }}_{L}\left(e-2,\;c,\;p-1,\;i-2\right)+{H^{\prime }}_{L}\left(e-1,\;c,\;p-1,\;i-1\right),\\ {H^{\prime }}_{L}\left(e-2,c-1,p,i\right)+\left(e-2-i\right)\cdot {H^{\prime }}_{L}\left(e-2,c,p,i\right)+ \end{array} & \left(3\right)\\ \;\;i\cdot {H^{\prime }}_{L}\left(e-2,\;c,\;p,\;i-1\right)+{H^{\prime }}_{L}\left(e-1,\;c,\;p,\;i\right), & \left(4\right)\\ \begin{array}{c} \left(e-1-i\right)\cdot {H^{\prime }}_{L}\left(e-2,\;c,\;p,\;i\right)+\\ \;\;i\cdot {H^{\prime }}_{L}\left(e-2,\;c,\;p-1,\;i-1\right)+{H^{\prime }}_{L}\left(e-1,\;c,\;p-1,\;i\right), \end{array} & \begin{array}{c} \left(5\right) \end{array} \end{array}\right)$$

with (1) if any of *e, c, p, i *is negative, or *e *= 0 and any of *c, p, i *is positive; (2) if *e *= *c *= *p *= *i *= 0; (3) if *e *= *i *> 0, (4) if *e *+ *i *is even, *e *>*i*, (5) if *e *+ *i *is odd, *e *>*i*.

#### Identity genome $I_{\ell_{i}}$ and input genomes $A_{\ell_{a}}$ with ℓ_i _and ℓ_a _linear chromosomes

In this scenario, in addiction to fixing *ℓ_i _*linear chromosomes for the identity $I_{\ell_{i}}$, we also build breakpoint graphs only with genomes $A_{\ell_{a}}$ that have exactly *ℓ_a _*linear chromosomes. We propose the Hultman number

(4) $$H_{\ell }\left(n,\;c,\;p,\;\ell_{i},\;\ell_{a}\right)\equiv |\left\{A_{\ell_{a}}\in G_{n,\ell_{a}}:cyc\left(BG\left(A,\;I_{\ell }\right)\right)=c\;\text{and}\;pt\left(BG\left(A,\;I_{\ell }\right)\right)=p\right\},$$

were $G_{n,\ell_{a}}$ is the set of all multichromosomal genomes with *n *genes and exactly *ℓ_a _*linear chromosomes, and *I_ℓ _*is, as before, a genome with exactly *ℓ *linear chromosomes. By definition, we have that $\sum_{\ell_{a}=0}^{n}H_{\ell }\left(n,\;c,\;p,\;\ell_{i},\;\ell_{a}\right)=H_{L}\left(n,\;c,\;p,\;\ell_{i}\right)$.

Again we define an auxiliary series, in this case $H_{\ell }^{\prime }\left(e,\;c,\;p,\;i,\;a\right)\equiv |\left\{M\in M_{e,\;a}:cyc\left(BG\left(M,\;M_{e,\;i}\right)\right)=c\;\text{and}\;pt\left(BG\left(M,\;M_{e,\;i}\right)\right)=p\right\}|$, where $M_{e,\;a}$ is the set of all possible matchings on *e *vertices that has exactly *a *unsaturated vertices, and $M_{I_{i}}$ is a matching on these vertices such that exactly *i *vertices are unsaturated. To build the breakpoint graph for this new series, we use exactly the same operations as in the previous, summarized in Figure 4. The only difference is that we have to track how many unsaturated vertices *a *the current matching being build has. The only operations that change this are the *skip vertex *operations (c), (i) and (f), decreasing *a *by 1. The other operations keep *a *the same, as they all create an edge and do not mark any vertex as unsaturated.

The base cases are also similar, only including *a *in the constraints. When *e *= 0 we have $H_{\ell }^{\prime }\left(0,\;c,\;p,\;i,\;a\right)=1$ if and only if *c *= *p *= *i *= *a *= 0, and $H_{\ell }^{\prime }\left(0,\;c,\;p,\;i,\;a\right)=0$ if any of *c, p, i, a *is positive. Also, if any of *e, c, p, i, a *is negative, $H_{\ell }^{\prime }\left(e,\;c,\;p,\;i,\;a\right)=0$.

Therefore, the recurrence is given by

$$H_{\ell }^{\prime }\left(e,\;c,\;p,\;i,\;a\right)=\left\{\begin{array}{cc} 0, & \left(1\right)\\ 1, & \left(2\right)\\ \begin{array}{c} \left(i-1\right)\cdot {H^{\prime }}_{\ell }\left(e-2,\;c,\;p-1,\;i-2,\;a\right)+\\ \;\;{H^{\prime }}_{\ell }\left(e-1,\;c,\;p-1,\;i-1,\;a-1\right), \end{array} & \begin{array}{c} \left(3\right) \end{array}\\ \begin{array}{c} {H^{\prime }}_{\ell }\left(e-2,\;c-1,\;p,\;i\right)+\left(e-2-i,\;a\right)\cdot {H^{\prime }}_{\ell }\left(e-2,\;c,\;p,\;i,\;a\right)+\\ \;\;i\cdot {H^{\prime }}_{\ell }\left(e-2,\;c,\;p,\;i-1,\;a\right)+{H^{\prime }}_{\ell }\left(e-1,\;c,\;p,\;i,\;a-1\right), \end{array} & \begin{array}{c} \left(4\right) \end{array}\\ \begin{array}{c} \left(e-1-i\right)\cdot {H^{\prime }}_{\ell }\left(e-2,\;c,\;p,\;i,\;a\right)+\\ \;\;i\cdot {H^{\prime }}_{\ell }\left(e-2,\;c,\;p-1,\;i-1,\;a\right)+{H^{\prime }}_{\ell }\left(e-1,\;c,\;p-1,\;i,\;a-1\right), \end{array} & \begin{array}{c} \left(5\right) \end{array} \end{array}\right)$$

with (1) if any of *e, c, p, i *is negative, or *e *= 0 and any of *c, p, i *is positive; (2) if *e *= *c *= *p *= *i *= 0; (3) if *e *= *i *> 0, (4) if *e *+ *i *is even, *e *>*i*, (5) if *e *+ *i *is odd, *e *>*i*.

## Distribution of rearrangement distances

From the Hultman series that we introduced, it is possible to derive the distribution of rearrangement distances for each scenario.

**Figure 5:**
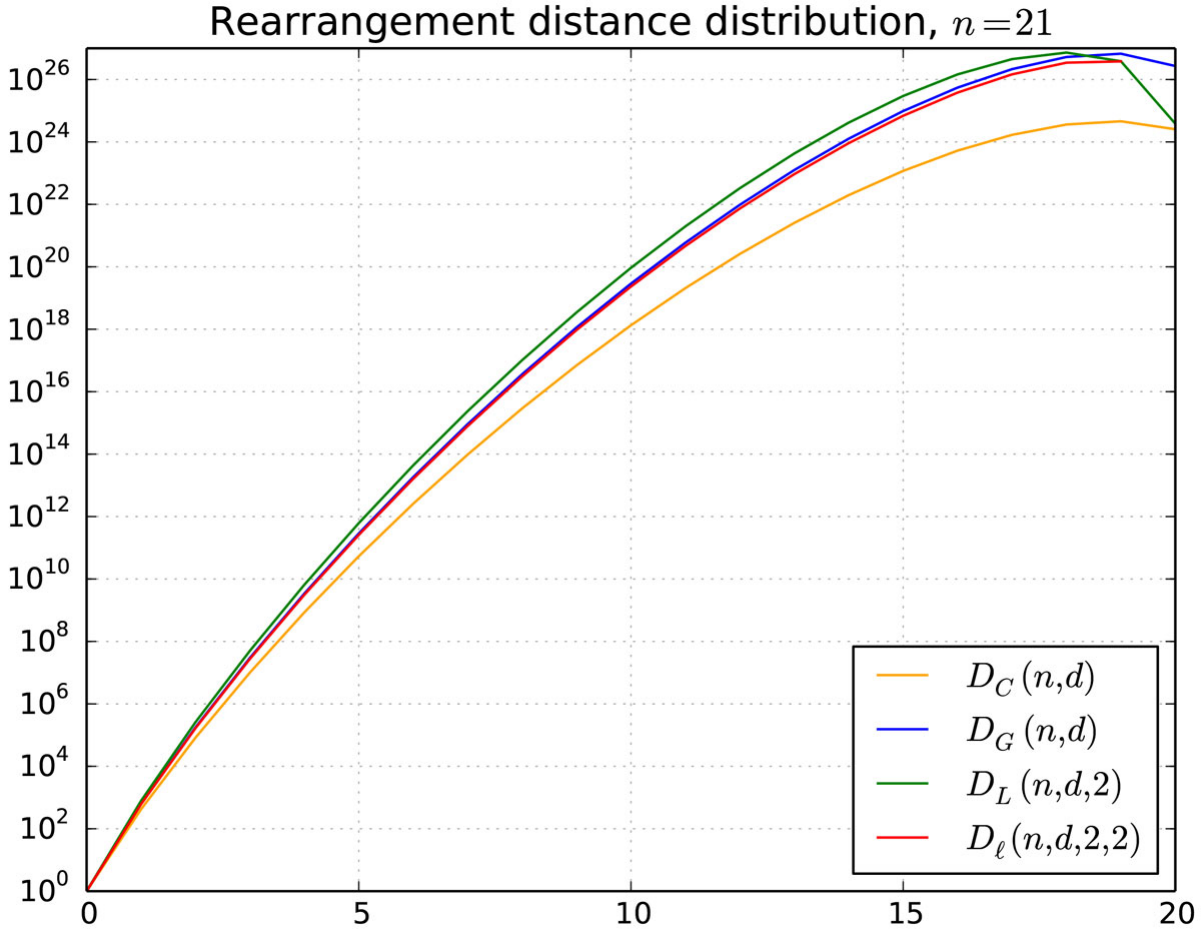
**Distribution of the rearrangement distance between genomes of size *n *= 21, in four different scenarios**.

The Double Cut and Join (DCJ) distance [9,10] is one of the most studied rearrangement distances since its introduction in 2005, because it can model several rearrangement operations and it is commonly easy to calculate in many cases. The DCJ distance between two genomes *A *and *B *is given by *d*(*A, B*) = *n - c - e*/2, where *n *is the number of genes, and *c *and *e *are respectively the number of cycles and *even *paths (paths with even number of edges) in the breakpoint graph *BG*(*A, B*). Using group theory, an alternative measure called *algebraic rearrangement distance *was proposed by Feijäo and Meidanis [11]. This distance can also be calculated with the breakpoint graph, namely *d_a_*(*A, B*) = *n - c - p*/2, where *n *is the number of genes, and *c *and *p *are respectively the number of cycles and paths in the breakpoint graph *BG*(*A, B*). Since the parity of paths is not important in the algebraic distance, it is the best suited model for calculating the distribution of the rearrangement distances from the Hultman numbers proposed here. For each of the four cases, we ask the following question: How many genomes of size *n *have distance *d *from a given identity genome? Making the same assumptions about the identity and also the universe of the genomes - that is, circular only, general, or a fixed number of linear chromosomes -, we arrive in the following distance distributions, shown also in Figure 5. It is interesting to notice that most of the genomes are very distant from the identity.

$$\begin{array}{c} D_{C}\left(n,\;d\right)\equiv |\left\{A\in C_{n}:d_{a}\left(A,\;I_{\circ }\right)=d\right\}|=H_{C}\left(n,\;n-d\right),\\ D_{G}\left(n,\;d\right)\equiv |\left\{A\in G_{n}:d_{a}\left(A,\;I_{\circ }\right)=d\right\}|=\underset{c+p/2=n-d}{\sum }H_{G}\left(n,\;c,\;p\right),\\ D_{L}\left(n,\;d,\;\ell \right)\equiv |\left\{A\in G_{n}:d_{a}\left(A,\;I_{\ell }\right)=d\right\}|=\underset{c+p/2=n-d}{\sum }H_{L}\left(n,\;c,\;p,\;\ell \right),\\ D_{\ell }\left(n,d,\;\ell_{i},\;\ell_{a}\right)\equiv |\left\{A_{\ell_{a}}\in G_{n,\;\ell_{a}}:d_{a}\left(A,\;I_{\ell }\right)=d\right\}|=\underset{c+p/2=n-d}{\sum }H_{\ell }\left(n,\;c,\;p,\;\ell_{i},\;\ell_{a}\right). \end{array}$$

Using those equations, we can also calculate the expected value for the rearrangement distance in any selected scenario. For instance, if we have the random variable *X_n _*= *d_a_*(*A_n_, I_n_*), where *I_n _*is the circular identity of size *n *and *A_n _*is a genome sampled uniformly from the set *C_n _*of all circular genomes, then we have

$$P\left[X_{n}=d\right]=\frac{D_{C}\left(n,\;d\right)}{\left|C_{n}\right|}=\frac{D_{C}\left(n,d\right)}{\left(2n-1\right)!!},$$

since |*C_n_*| is the number of circular genomes of size *n *and corresponds to the number of perfect matchings with 2*n *vertices, given by (2*n - *1)!!. The expected value is then given by

$$E\left[X_{n}\right]=\overset{n}{\underset{d=0}{\sum }}d\cdot P\left[X_{n}=d\right]=\frac{1}{\left(2n-1\right)!!}\overset{n}{\underset{d=0}{\sum }}d\cdot H_{C}\left(n,\;n-d\right),$$

and can therefore be calculated with the given recurrence equations. For instance, for *n *= 100 we have *E*[*X*_100_] = 95.22. A closed formula for the expected value of a rearrangement distance, to the best of our knowledge, has only been found for the very simple *breakpoint distance d_BP_*, which counts how many adjacent genes in the identity are not adjacent in the other genome, and is given by $E\left[d_{BP}\left(A_{n},\;I_{n}\right)\right]=n-\left(\frac{1}{2}+\frac{1}{2n}+O\left(\frac{1}{n^{2}}\right)\right)$[12]. This converges to *n *- 1/2 when *n *goes to infinity, which is almost the diameter *n *for the breakpoint distance. Although we have no closed formula for *E*[*X_n_*], we conjecture that it also converges to *n - k *for some constant *k *> 0, as *n *goes to infinity, and the experimental results point to *k *≈ 5.

## Conclusions

In this paper, we introduced different recursive formulas for the Hultman number and its variations, that are relevant in the context of comparative genomics. We have extended previous results that treated the unichromosomal cases [3,4], focusing on multichromosomal genomes. Table 1 shows a summary of the results.

**Table 1:** Summary of the results in this paper.

Hultman Number	Identity	Universe
H(n,k)[3]	*π *= ⟨ ⋯ ⟩	*S_n _*(unsigned permutations)
$H^{\pm }\left(n,\;k\right)$[4]	*π *= ⟨ ⋯ ⟩	$S_{n}^{\pm }\left(\text{signed}\;\text{permutations}\right)$
*H_C _*(*n, c*)	Circular genome	Circular genomes
*H_G_*(*n, c, p*)	Circular genome	General genomes
*H_L_*(*n, c, p, ℓ*)	Genome with *ℓ *linear chr.	General genomes
*H_ℓ _*(*n, c, ℓ_i_, ℓ_a_*)	Genome with *ℓ_i _*linear chr.	Genomes with *ℓ_a _*linear chr.

The first two rows show previous results, and the last four show the Hultman numbers proposed in this paper.

For the Hultman number *H_C _*(*n, c*), in addition to the recursive equations we also provided an explicit formula, using the relationship between this series and the unsigned Stirling numbers of first kind. An interesting future direction is finding explicit formulas for the other proposed sequences *H_G_*(*n, c, p*) and *H_L_*(*n, c, p, ℓ*).

Another interesting relationship is that, for a fixed *n*, the sum of all combination of cycles and paths in a series results in the number of genomes of size *n*. The number of circular genomes of size *n *corresponds to the number of perfect matchings with 2*n *vertices, which is given by (2*n - *1)!!. The number of general genomes of size *n *is the number of matchings with 2*n *vertices, which is the *telephone number T *(*n*) (sequence A000085 in OEIS [8]), given by $T\left(n\right)=\sum_{k=0}^{\left\lfloor n/2\right\rfloor }\frac{n!}{2^{k}\left(n-2k\right)!k!}$. The equations below follow:

$$\overset{n}{\underset{c=0}{\sum }}H_{C}\left(n,\;c\right)=\left(2n-1\right)!!,\;\;\overset{n}{\underset{c=0}{\sum }}\overset{n}{\underset{p=0}{\sum }}H_{G}\left(n,\;c,\;p\right)=T\left(n\right),$$

$$\overset{n}{\underset{c=0}{\sum }}\overset{n}{\underset{p=0}{\sum }}H_{L}\left(n,\;c,\;p,\;\ell \right)=T\left(n\right),\;\;\text{for}\;\ell =0,\;\ldots ,\;n.$$

and

$$\overset{n}{\underset{\ell_{a}=0}{\sum }}\overset{n}{\underset{c=0}{\sum }}\overset{n}{\underset{p=0}{\sum }}H_{\ell }\left(n,\;c,\;p,\;\ell_{i},\;\ell_{a}\right)=T\left(n\right),\;\;\text{for}\;\ell_{i}=0,\;\ldots ,\;n.$$

These equations might be useful for finding explicit equations for some of the numbers. We wrote a Python script with all recurrence relations proposed, and the above equations were useful to check the correctness of each series.

The Hultman number can also be used to find the expected value of the rearrangement distance between uniformly distributed genomes, in our case the algebraic distance between multichromosomal genomes. Future directions include finding explicit equations for the introduced recursive equations and the expected value of the rearrangement distance.

## Competing interests

The authors declare that they have no competing interests.

## Authors' contributions

All authors contributed equally.
